# Are Maternal Feeding Practices and Mealtime Emotions Associated with Toddlers’ Food Neophobia? A Follow-Up to the DIT-Coombe Hospital Birth Cohort in Ireland

**DOI:** 10.3390/ijerph17228401

**Published:** 2020-11-13

**Authors:** Meijing An, Qianling Zhou, Katherine M. Younger, Xiyao Liu, John M. Kearney

**Affiliations:** 1 Department of Maternal and Child Health, School of Public Health, Peking University, 38 Xueyuan Road, Haidian District, Beijing 100191, China; mj_an1128@bjmu.edu.cn (M.A.); liuxiyao@bjmu.edu.cn (X.L.); 2 School of Biological Sciences, Technological University Dublin, Kevin Street Dublin 8, D08 X622 Dublin, Ireland; katherine.younger@TUDublin.ie (K.M.Y.); john.kearney@TUDublin.ie (J.M.K.)

**Keywords:** toddlers, food neophobia, feeding practices, mealtime emotions, food refusal

## Abstract

This study was conducted to explore the associations between maternal feeding practices, mealtime emotions, as well as maternal food neophobia and toddlers’ food neophobia in Ireland. A follow-up to the Technological University Dublin (DIT)-Coombe Hospital birth cohort was conducted. Mothers in the original cohort were invited to the present study by telephone calls. Postal questionnaires with stamped addressed envelopes were distributed to those who agreed to participate in the study. Toddler food neophobia was assessed by the modified version of the Child Food Neophobia Scale (CFNS). There were 205 participants included in this study, with a median score of child food neophobia of 12. A higher degree of child food neophobia (score > 12) was positively associated with the maternal practice of coaxing the children to eat at refusal (OR (Odds Ratio) = 2.279, 95% CI: 1.048–4.955), unpleasant emotions at mealtime (e.g., stressful or hectic for mothers, or tearful for children) (OR ranged between 1.618 and 1.952), and mothers’ own degree of food neophobia (OR = 1.036, 95% CI: 1.001–1.072). Mothers who were not worried when confronted with child’s food refusal was negatively associated with toddlers’ food neophobia (OR = 0.251, 95% CI: 0.114–0.556). This study suggests the maternal practices of responsive feeding, being calm and patient with the toddlers, and creating a positive atmosphere at mealtime.

## 1. Introduction

Toddlerhood is a crucial period for the development of eating habits, which normally track into adulthood [1]. Food neophobia is regarded as the reluctance to eat, or the avoidance of new foods [2]. It starts at about two years of age, and peaks at two to six years of age [2]. From the evolutionary perspective, food neophobia helps toddlers, who begin to walk and explore their environment, to avoid toxic foods [2]. However, in modern society, foods are generally safe to eat; the protective effects of food neophobia have thus become less valuable [1]. Moreover, food neophobia is a common barrier to the acquisition of healthy eating habits during toddlerhood [3,4]. A study has shown that children aged one to five years who have a higher degree of food neophobia are more likely to have poor dietary quality [5]. Compared to children without food neophobia, those with food neophobia consume less fruits and vegetables [6,7], meat [7] and fish [1].

Mothers are major care providers of children. Maternal feeding practices, mealtime emotions, and maternal food neophobia, might be associated with children’s food neophobia [3,8,9]. Existing studies have focused on feeding practices. For instance, a study conducted among three to twelve year old children showed that mothers with children high in food neophobia used more controlling feeding strategies [9]. Both studies conducted in children aged three to five years [8] and children aged one to seven years [10] reported that maternal pressuring to eat was associated with children’s food neophobia. Studies about children’s food neophobia have focused on preschoolers and older children. Fewer studies are conducted among toddlers individually. Studies in Australia [11], the United States [12] and France [13] found that maternal coercive feeding practices were positively associated with toddlers’ food neophobia, while autonomy-supportive prompts were negatively associated with toddlers’ food neophobia. To our knowledge, no relevant investigation on toddlers’ food neophobia has been conducted in the Republic of Ireland. Nevertheless, the prevalence of breastfeeding in Ireland is among the lowest level globally [14], implying that toddlers’ food neophobia might be prevalent [15]. As a result, it is necessary to explore toddlers’ food neophobia and its risk factors in Ireland.

Family mealtimes provide opportunities to express emotions between mother and children. Mealtime emotions were related to parent feeding and children’s food consumption [16,17]. However, mealtime emotions were not examined in the above studies about maternal feeding practices. A study in Australia found higher maternal concern about infant under-eating was associated with higher child food neophobia at 24 months [11]. Relevant studies are needed in other countries to understand the cultural variations of the findings.

This study was conducted to fill an information gap, in examining the associations between maternal feeding practices and mealtime emotions and toddler’s food neophobia in the Republic of Ireland. 

## 2. Materials and Methods 

### 2.1. Study Design and Population

This was a follow up to the Technological University Dublin (DIT)-Coombe Hospital study [18], which was designed to examine the prevalence and determinants of breastfeeding initiation and duration, and weaning practices in Ireland. The DIT-Coombe Hospital study was a birth cohort conducted between June 2004 and October 2006 among 520 mothers (87.3% were born in Ireland) who gave birth in the Coombe Women and Infants University Hospital in Dublin [18,19]. The current follow-up study was conducted from September 2007 to January 2008, to examine maternal feeding practices, mealtime emotions and toddlers’ eating behaviors. All mothers in the birth cohort were contacted via telephone calls, and were invited to take part in the current study. Four hundred and forty-eight mothers were contactable, and 444 agreed to participate. A postal questionnaire with a stamped address envelope was then delivered to those 444 mothers. Mothers were required to fill the consent form and the questionnaire, and return them by post.

### 2.2. Children and Maternal Socio-Demographic Covariates

Participants’ general characteristics were extracted from the original cohort data. The variables included mother’s age at time of childbirth, marital status, education, occupation, accommodation, health insurance status, children’s date of birth, birthplace, breastfeeding initiation and child’s age of weaning onto solid. The present study (i.e., follow up study) included questions related to food neophobia of children and mothers, maternal toddler feeding practices when the child refused to eat or was slow to eat, maternal mealtime emotions with the child, and the date of completing the current questionnaire.

### 2.3. Child Food Neophobia

Children’s food neophobia was assessed by the 6-item Child Food Neophobia Scale (CFNS, Table 1), which has been used in previous studies and confirmed good readability and acceptance [6,20]. Response was based on a 4-point Likert scale from “strongly disagree” to “strongly agree”. Cronbach’s alpha of the scale was 0.917 in the present sample, indicating good internal reliability. The total score ranged from six to 24. Higher scores indicate a higher degree of food neophobia. The median value of CFNS had been used to differentiate children with high or low level of food neophobia [3,6]. In the present study, children were divided into a high food neophobia group (score > 12) and a low food neophobia group (score ≤ 12) according to the median value (12) of the modified CFNS. 

### 2.4. Maternal Food Neophobia

Maternal food neophobia was assessed by the modified version of the adult Food Neophobia Scale (FNS). The scale was modified by the research team, and included nine items of the original FNS. The items “at dinner parties, I will try a new food” and “I like to try new ethnic restaurants” in the original scale [21] were deleted, and the item “I prefer to eat foods I am used to” was added (see Table A1 in Appendix A). Response to the modified FNS was based on a 7-point Likert scale, ranging from “strongly disagree” to “strongly agree”. The total score ranged from nine to 63. Higher scores indicate greater maternal food neophobia. The modified FNS was pilot tested and confirmed good readability and acceptance. Cronbach’s alpha of modified FNS was 0.824 of the present sample, indicating good internal reliability of the modified scale. 

### 2.5. Maternal Feeding Practices and Mealtime Emotions

Maternal feeding practices and mealtime emotions with child were assessed by a series of questions adopted from the literature [22] with some modification. The questions included “If your child refuses to eat, do you tend to take it away, coax her/him to eat, punish her/him for not eating, or not worry too much about it? (mark ‘Yes’ or ‘No’)”, “If your child is slow to eat or finish a meal, do you try to distract her/him during eating (e.g., playing aeroplanes, watching TV, etc.)? (choose from ‘never’ to ‘almost all the time’)”, as well as mealtime emotions (including “relaxed to stressful for mother”, “unrushed to hectic for mother”, and “happy to tearful for your child”, scored from 1 to 5 for each domain).

### 2.6. Data Analysis

Continuous variables were described as Mean ± SD, and categorical variables were described as counts and percentages. Univariate analyses (including the Pearson chi-square test and independent-samples *t* test) were performed to examine the differences of children’s food neophobia score across participants’ general characteristics and maternal feeding practices and emotions. Logistic regression analysis was further performed to determine the association between children’s high degree of food neophobia and maternal feeding practices/emotions after adjusting for potential confounders. Two regression models were constructed. In Model 1, the relationships between each independent variable and children’s food neophobia were assessed, with the adjustment of child’s age. In Model 2, when “mealtime emotions (V3)” was treated as a covariate, to avoid collinearity, scores of three items were summed up as total V3 score. Total V3 score was categorized into two groups (total score < 8 and ≥8) as a covariate (total V3). When items in V1 (maternal practices/emotions when child refuses to eat) were treated as covariates, coaxing to eat and not worrying about it too much when child refuses to eat were included into the model. In Model 2, V1, V2 (mother trying to distract the child when the child is slow to eat or finish a meal), V3 and V4 (maternal FNS) were controlled for each other besides child’s age. SPSS statistical software (version 22.0) was used in the data analyses, and the statistically significant level was set as *p* < 0.05.

## 3. Results

### 3.1. Socio-Demographic Characteristics of Participants

A total of 209 mothers returned the questionnaire by post. Compared with those who did not respond to the survey, respondents were more likely to be older, married, have a higher education level, have a professional occupation, live in their own property, have private health insurance, have initiated breastfeeding, and have weaned their baby onto solid to more than 12 weeks after birth (Table A2 in Appendix A). With a further exclusion of participants who failed to answered questions related to child food neophobia (*n* = 4), 205 participants were included in final data analysis. Of the 205 children, mean age was 2.43 ± 0.71 years (ranged 1.5–3.42 years). There were 115 (56.1%) boys; 123 (60%) children were breastfed, and 175 (85.4%) were weaned onto solid more than 12 weeks after birth.

### 3.2. Children’s Food Neophobia Score

Table 1 presents the descriptive statistics regarding children’s food neophobia score. The median was 12. Children with a high food neophobia score were older than their counterparts with a low score (2.7 ± 0.7 v.s. 2.2 ± 0.7 years, *p* < 0.001). Maternal socio-demographic variables, type of delivery, breastfeeding and weaning practices had no significant relationships with children’s food neophobia score (*p* ranged 0.108–0.965, Table 2). 

### 3.3. Univariate Analysis Results

Table 3 shows the relationships between maternal toddler feeding factors and children’s degree of food neophobia, by univariate analyses. When children refused to eat, the mother’s behavior of coaxing children to eat (*p* < 0.001), punishing children for not eating (*p* = 0.037), and being worried about children’s refusal (*p* < 0.001) were associated with children’s high food neophobia score. If the child was slow to eat, mothers who distracted children during eating quite often/almost all the time (*p* = 0.022) tended to have children with a higher food neophobia score. At mealtime, mothers feeling stressful (*p* < 0.001), hectic (*p* < 0.001), and tearful for the child (*p* < 0.001) was significantly associated with higher children’s food neophobia score. A maternal food neophobia score was marginally associated with a high children’s food neophobia score (*p* = 0.051). 

### 3.4. Logistic Regression Analysis Results

The independent effects of maternal feeding practices and emotion on children’s food neophobia were assessed, after controlling for potential confounders (Table 4). As shown in Model 1 and Model 2, maternal having unpleasant feeling at mealtime, such as stressful or hectic for themselves, tearful for the children, as well as maternal food neophobia scores were positively related to high children’s food neophobia scores after controlling for maternal feeding practices. In Model 1, frequent use of distraction strategies if a child was slow to eat was associated with a higher children’s food neophobia score, in comparison with no use of distraction (OR (Odds Ratio) = 2.982, 95% CI: 1.134–7.842). In Model 2, when children refused to eat, the maternal practice of coaxing them to eat was associated with a higher children’s food neophobia score (OR = 2.279, 95% CI: 1.048–4.955) after controlling for mealtime emotions; while the mothers not worrying too much was negatively related to the degree of children’s food neophobia (OR = 0.251, 95% CI: 0.114–0.556) after controlling for maternal feeding practices. 

## 4. Discussion

This study provided evidence about the relationships between toddlers’ food neophobia and maternal feeding practices, mealtime emotions and maternal food neophobia in Ireland. It was demonstrated that toddler’s food neophobia was positively associated with (a) the maternal feeding practice of coaxing the toddler to eat, and (b) mealtime emotions of maternal stressful and hectic feeling, toddler’s tearful expression at mealtime or maternal worrying too much when children refused to eat. Not surprisingly, maternal food neophobia was associated with toddler’s food neophobia.

Children’s food refusal can be confusing to parents because mothers might be unclear whether children are signaling satiety, requesting an alternative, or exhibiting a behavioral problem [23]. Parents are generally concerned about children’s capability to determine satiety, and the health consequences of not having an adequate amount of food [23]. Most parents have no solution to children’s food refusal other than force-feeding [23]. Our study reported that maternal coaxing of the toddler to eat at food refusal was positively related to higher children food neophobia. Such a finding was consistent with Cassells et al. [11] and Hebah [10] that maternal coercive eating strategies such as pressure to eat were common among children who expressed food neophobia. Parental prompting to eat was found to be associated with toddler’s food refusal [12]. In fact, young children are capable of regulating internal hunger and satiety signals [23], and responsive feeding is an optimal child feeding practice. If the mother forces the child to eat, the child’s internal regulatory ability may be disordered, which in turn affect the child’s feeling about foods and his/her control of satiety. Batsell et al. [24] stated that children being forced to consume certain foods would develop a “cognitive aversion” for those foods due to the negative feeding experience, which could be a catalyst for food neophobia [25]. A longitudinal study reported that girls who received a high level of pressure to eat from their mothers at age seven years would have fussy eating behaviors at nine years of age [26]. On the contrary, offering children new foods in an non-coercive manner could reduce children’s reluctance to new foods [8] and children’s low food refusal [12]. 

In the present study, if a child was slow to eat or finish a meal, the mothers’ use of distraction strategy was positively associated with the child’s food neophobia. Our result was supported by the literature. Powell found that no distractors (e.g., no toys, TV or magazines) at mealtimes was associated with less fussy eating behaviors of the children [27]. Distractors at mealtime were associated with a higher energy intake [28] and overweight [29] of children, and thus are recommended to be avoided. In our study, the association between maternal distraction strategies when the child is slow to eat or finish a meal and children’s food neophobia was not significant. Owing to the limited amount of evidence in the literature, evidence-based advice on child feeding needs delivered to the children.

Our study found that mothers not worrying too much when the child refused to eat was negatively related to children’s food neophobia. Such a finding was consistent with a previous study that stated that higher maternal concern about an infant under-eating and becoming underweight at four months was associated with higher child food neophobia at two years [11]. Our study demonstrated that mothers’ feeling stressful or hectic, or considering the child tearful at mealtimes were positively associated with children’s food neophobia. Mothers’ unpleasant feeling may be the results of misinterpretation of children’s expression such as slowdown. The slowdown might be caused by the abatement of hunger or a desire to explore the surroundings. Maternal misinterpretation of the slowdown as early food refusal might lead to mealtime stress for both mothers and the child [23]. As a result, mothers are recommended to be patient with children at mealtime and avoid unpleasant emotions such as stress and hectic behavior. Children’s tearful expression may derive the bad feeling towards foods such as disgust or may be the expression of food refusal. Studies have showed that disgust was related to food neophobia [30]. Food neophobia may indicate fear [31] and anxiety [32] toward new foods. Neophobic children were more likely to cry to refuse a food [12]. The current result was based on the cross-sectional data, and the direction of the relationship between mealtime emotions and children’s food neophobia needs to be confirmed by longitudinal studies. Since mealtime emotions are modifiable factors for children’s food consumption, study examining the role of maternal feeding practices together with mealtime emotions on children’s food neophobia might be useful in the development of comprehensive strategies to improve children’s healthy eating behaviors and food consumption.

Some evidence showed that there was familial resemblance of food neophobia [3,33,34]. Our study found that maternal food neophobia score was positively related to children’s food neophobia score. Our finding was in consistent with Kaar et al. [8] and Finistrella et al. [3] among American preschooler-parent dyads and Italian preschooler-mother dyads, respectively. The correlation of food neophobia between mother and child could be explained by heritability (estimated 66–78%) [35,36], as well as temperamental traits and personality factors [37,38]. Moreover, neophobic mothers might be more likely to judge an infant’s displays as disliking of foods and stop offering the foods [39]. Mothers with food neophobic children and mothers who were themselves food neophobic reported that they do not make healthy foods readily available for their children [9]. In addition, Moding et al. found that parents with neophobia increased their tendency to describe their children as neophobic, even among children who were rated by observers as low in behavioral neophobia [40]. As a result, objective measures such as video-recording to assess children’s eating behaviors and food neophobia might be considered in future studies.

This study adds evidence about relationships between maternal feeding practices and mealtime emotions and toddlers’ food neophobia in Ireland. However, there are some limitations to be acknowledged. First, owing to its cross-sectional nature, the causal relationships cannot be established. Whether children’s food neophobia elicits more inappropriate maternal feeding practices or unpleasant emotions at mealtimes, or whether maternal feeding factors foster children’s food neophobia need to be explored in longitudinal studies further. Otherwise, intervention studies should be designed to explore whether managing maternal feeding practices and mealtime emotions result in improvement in neophobia in offspring and to help parents understand the importance of feeding practices and mealtime emotions for reducing offspring’s food refusal. Second, this was a follow up to a birth cohort study. The follow up rate was relatively low. The time interval and postal survey might account for this. Third, all information (e.g., mother and children’s food neophobia, mother–child mealtime interactions and emotions) was reported by mothers. Respondent bias and social desirable bias were thus inevitable. Finally, some feeding practices such as restriction, food as a reward and controlling practices were not examined in this study, and should be considered in future studies. 

## 5. Conclusions

Our study added novel evidence regarding the relationships between maternal feeding practices and mealtime emotions and toddler’s food neophobia in Ireland. Maternal coaxing of children to eat and worrying too much when their child refuses to eat were positively associated with children’s food neophobia. Mothers’ feeling at mealtime with children (e.g., stressful, tearful) was related to children’s food neophobia. Future longitudinal studies can be expected to illustrate the direction of such relationships. To enhance children’s acceptance of new foods, caregivers adopting responsive feeding strategies, being patient with children and avoiding unpleasant feeling at mealtime may be helpful.

## Figures and Tables

**Table 1:** Assessment of children’s food neophobia (*n* = 205).

Variables	Score Range	Values
Modified CFNS Items		(Mean ± SD)
My child is constantly sampling new and different foods (R)	1–4	2.14 ± 0.76
My child is afraid to eat things she/he has never had before	1–4	2.14 ± 0.80
My child does not trust new foods	1–4	2.13 ± 0.76
If my child does not know what is in a food, she/he will not try it	1–4	2.03 ± 0.84
My child eats anything (R)	1–4	2.44 ± 0.87
My child is very particular about the foods she/he will eat	1–4	2.15 ± 0.88
Total score of the Modified CFNS	6–24	13.02 ± 4.15
Median (IQR) of the Modified CFNS		12 (11,15)
**Children’s food neophobia groups**		***n*** **(%)**
Low children’s food neophobia score (score ≤ 12)		107 (52.2)
High children’s food neophobia score (score > 12)		98 (47.8)

CFNS, Children’s Food Neophobia Scale; IQR, interquartile range; R, score counted reversely.

**Table 2:** Characteristics and infant feeding practices of participants classified by participants with low and high children’s food neophobia scores.

Variables	All Participants	Participants with a Low Children’s Food Neophobia Score	Participants with a High Children’s Food Neophobia Score	*χ^2^/t*	*p*
	(*n* = 205)	(*n* = 107)	(*n* = 98)		
	*n* (%) or Mean ± SD				
Child’s age	2.4 ± 0.7	2.2 ± 0.7	2.7 ± 0.7	−4.336	<0.001
Child’s gender					
Male	115	60(52.2)	55(47.8)	<0.001	0.995
Female	90	47(52.2)	43(47.8)		
Mother’s age at time of childbirth					
15–24 years old	20	11(55.0)	9(45.0)	0.070	0.965
25–34 years old	129	67(51.9)	62(48.1)		
>34 years old	56	29(51.8)	27(48.2)		
Maternal marital status					
Married	160	84(52.5)	76(47.5)	0.027	0.869
Single/divorced/widow	45	23(51.1)	22(48.9)		
Maternal education					
Primary/Secondary level	59	29(49.2)	30(50.8)	0.307	0.858
Vocational/training course	58	31(53.4)	27(46.6)		
Third level including post graduate	88	47(53.4)	41(46.6)		
Maternal occupation					
Professional/Managerial/Technical Workers	80	41(51.2)	39(48.8)	2.690	0.442
Non-Manual	57	33(57.9)	24(42.1)		
Skilled Manual/Semi-Skilled	18	11(61.1)	7(38.9)		
Students/Unemployed/Housewife	50	22(44.0)	28(56.0)		
Accommodation					
Home/Apartment owner	172	94(54.7)	78(45.3)	2.583	0.108
Non-home owners	33	13(39.4)	20(60.6)		
Health insurance status					
Public	85	44(51.8)	41(48.2)	0.306	0.858
Semi-private	77	39(50.6)	38(49.4)		
Private	43	24(55.8)	19(44.2)		
Maternal birthplace					
Republic of Ireland	179	92(51.4)	87(48.6)	0.361	0.548
Countries outside Ireland	26	15(57.7)	11(42.3)		
Type of delivery					
Spontaneous Vaginal Delivery	151	77(51.0)	74(49.0)	0.332	0.565
Caesarean Section	54	30(55.6)	24(44.4)		
Had initiated breastfeeding					
Yes	123	64(52.0)	59(48.0)	0.003	0.954
No	82	43(52.4)	39(47.6)		
Child’s age of weaning onto solid					
≤12 weeks	30	16(53.3)	14(46.7)	0.018	0.893
>12 weeks	175	91(52.0)	84(48.0)		

Pearson chi-square test for categorical variables and independent-samples *t* test for continuous variables.

**Table 3:** Maternal toddler feeding factors, classified by participants with low and high children’s food neophobia scores.

Variables	All Participants	Participants with a Low Children’s Food Neophobia Score	Participants with a High Children’s Food Neophobia Score	*χ^2^/t*	*p*
	(*n* = 205)	(*n* = 107)	(*n* = 98)		
	*n* (%) or Mean ± SD				
Does the child usually eat with family members?					
Yes, most of the time	185	100(54.1)	85(45.9)	2.626	0.105
No, not usually	20	7(35.0)	13(65.0)		
If your child refuses to eat, you tend to					
Take it away					
No	115	54(47.0)	61(53.0)	2.689	0.101
Yes	78	46(59.0)	32(41.0)		
Coax her/him to eat					
No	58	41(70.7)	17(29.3)	12.523	<0.001
Yes	133	57(42.9)	76(57.1)		
Punish her/him for not eating					
No	186	98(52.7)	88(47.3)	4.353	0.037
Yes	4	0(0.0)	4(100.0)		
Not worry too much about it					
No	63	18(28.6)	45(71.4)	21.801	<0.001
Yes	134	86(64.2)	48(35.8)		
If your child is slow to eat or finish a meal, do you try to distract her/him during eating (e.g., playing aeroplanes, watching TV, etc.)?					
Never	51	28(54.9)	23(45.1)	7.619	0.022
Sometimes	121	69(57.0)	52(43.0)		
Quite often/Almost all the time	33	10(30.3)	23(69.7)		
How you feel at mealtimes with your child?					
Stressful for you	2.36 ± 1.07	1.95 ± 0.89	2.80 ± 1.07	−6.083	<0.001
Hectic for you	2.27 ± 1.11	1.98 ± 0.99	2.58 ± 1.15	−3.979	<0.001
Tearful for your child	1.76 ± 0.85	1.43 ± 0.66	2.12 ± 0.89	−6.237	<0.001
Maternal food neophobia score	27.96 ± 10.76	26.50 ± 10.06	29.51 ± 11.30	−1.964	0.051

Pearson chi-square test for categorical variables and independent-samples *t* test for continuous variables.

**Table 4:** Associations between maternal feeding factors and child’s high food neophobia score with controlling covariates.

Variables	Model 1	Model 2
V1: If your child refuses to eat, you tend to		
Take it away (Yes)	0.845 (0.450, 1.586)	0.869 (0.426, 1.775)
Coax her/him to eat (Yes)	2.787 (1.397, 5.559)	2.279 (1.048, 4.955)
Punish her/him for not eating (Yes)	NA	NA
Not worry too much about it (Yes)	0.202 (0.101, 0.407)	0.251 (0.114, 0.556)
V2: If your child is slow to eat or finish a meal, do you try to distract her/him during eating (e.g., playing aeroplanes, watching TV, etc.)?		
Never	1.000	1.000
Sometimes	1.027 (0.515, 2.046)	1.116 (0.460, 2.708)
Quite often/Almost all the time	2.982 (1.134, 7.842)	1.853 (0.528, 6.502)
V3: How you feel at mealtimes with your child?		
Stressful for you	2.338 (1.677, 3.258)	1.952 (1.292, 2.949)
Hectic for you	1.805 (1.348, 2.418)	1.618 (1.143, 2.291)
Tearful for your child	2.840 (1.862, 4.331)	1.783 (1.066, 2.981)
V4: Maternal food neophobia score	1.037 (1.008, 1.067)	1.036 (1.001, 1.072)

NA: Not available. When the V3 variable was the covariate, the total score of the V3 variables were computed by summing up three items due to collinearity. Then the total score was classified into two groups. One is total score < 8, the other is total score ≥ 8. Model 1 controlled for child’s age. In Model 2, for each item of V1, child’s age, V2, total V3 (dinominal) and maternal FNS were controlled; for V2, child’s age, coaxing to eat and not worrying about it too much when child refuses to eat, total V3 (dinominal) and maternal FNS were controlled; for each item of V3, child’s age, coaxing to eat and not worrying about it too much when child refuses to eat, V2 and maternal FNS were controlled; for V4, child’s age, coaxing to eat and not worrying about it too much when child refuses to eat, V2 and total V3 (dinominal) were controlled.

## Appendix A

**Table A1:** Assessment of maternal food neophobia (*n* = 195).

Variables	Score Range	Mean ± SD
Modified FNS items		
I am constantly sampling new and different foods (R)	1–7	2.76 ± 1.51
I do not trust new foods	1–7	2.37 ± 1.52
If I do not know what is in a food, I will not try it	1–7	3.91 ± 2.15
I like foods from different countries (R)	1–7	2.46 ± 1.73
Ethnic food looks too weird to eat	1–7	2.56 ± 1.83
I am afraid to eat things I have never had before	1–7	2.53 ± 1.79
I prefer to eat foods I am used to	1–7	3.90 ± 2.05
I am very particular about the foods I will eat	1–7	4.13 ± 2.10
I will eat almost anything. (R)	1–7	3.34 ± 2.02
Total score	9–63	27.96 ± 10.76
Median (IQR)	27 (20, 35)	

FNS, Food Neophobia Score; IQR, interquartile range; R, score counted reversely.

**Table A2:** Socio-demographic characteristics of participants of the Technological University Dublin (DIT)-Coombe Cohort study (*n* = 520), respondents (*n* = 209) and non-respondents (*n* = 311) of the current follow-up study.

Variables	Participants of the DIT-Coombe Cohort (*n* = 520)	Respondents to the Current Follow-Up Study (*n* = 209)	Non-Respondents to the Current Study (*n* = 311)
	*n* (%) or Mean ± SD		
Mother’s age at time of childbirth *			
15–24 years old	107 (20.6)	20 (9.6)	87 (28.0)
25–34 years old	306 (58.8)	132 (63.2)	174 (55.9)
>34 years old	107 (20.6)	57 (27.3)	50 (16.1)
Maternal marital status *			
Married	322 (61.9)	163(78.0)	159 (51.1)
Single/divorced/widow	198 (38.1)	46 (22.0)	152 (48.9)
Maternal education *			
Primary/Secondary level	200 (38.5)	61 (29.2)	139 (44.7)
Diploma	143 (27.5)	58 (27.8)	85 (27.3)
Third level	177 (34.0)	90 (43.1)	87 (28.0)
Maternal occupation *			
Professional/Managerial/Technical Workers	161 (31.0)	81 (38.8)	80 (25.7)
Non-Manual	145 (27.9)	57 (27.3)	88 (28.3)
Skilled Manual/Semi-Skilled	57 (11.0)	19 (9.1)	38 (12.2)
Students/Unemployed/Housewife	157 (30.2)	52 (24.9)	105 (33.8)
Accommodation *			
Home/Apartment owner	361 (69.4)	175 (83.7)	186 (59.8)
Non-home owners	159 (30.6)	34 (16.3)	125 (40.2)
Health insurance status *			
Public	277 (53.3)	87 (41.6)	190 (61.1)
Semi-private	169 (32.5)	78 (37.3)	91 (29.3)
Private	74 (14.2)	44 (21.1)	30 (9.6)
Maternal birthplace			
Republic of Ireland	454 (87.3)	183 (87.6)	271 (87.1)
Countries outside Ireland	66 (12.7)	26 (12.4)	40 (12.9)
Child’s age when the follow-up study was conducted (years)	2.42 ± 0.71	2.44 ± 0.71	2.41 ± 0.70
Child’s gender			
Male	283 (54.4)	116 (55.5)	167 (53.7)
Female	237(45.6)	93 (44.5)	144 (46.3)
Had initiated breastfeeding *			
Yes	262 (50.4)	124 (59.3)	138 (44.4)
No	258 (49.6)	85 (40.7)	173 (55.6)
Child’s age of weaning onto solid *			
≤12 weeks	106 (20.4)	30 (14.4)	76 (24.4)
>12 weeks	414 (79.6)	179 (85.6)	235 (75.6)

* *p* < 0.05, Pearson Chi square for categorical variables and independent *t* test for continuous variables, to compare the differences of socio-demographic characteristics between respondents and non-respondents in the current follow up study.
